# Genetic variation and structure of complete chloroplast genome in alien monoecious and dioecious *Amaranthus* weeds

**DOI:** 10.1038/s41598-022-11983-2

**Published:** 2022-05-18

**Authors:** Han Xu, Ning Xiang, Wei Du, Junhua Zhang, Yongjiang Zhang

**Affiliations:** 1 Institute of Plant Inspection and Quarantine, Chinese Academy of Inspection and Quarantine, Beijing, 100176 China; 2 Agricultural Technology Extension Station of Ningxia, Yinchuan, 750001 China

**Keywords:** Evolution, Genetics, Molecular biology, Plant sciences, Systems biology

## Abstract

*Amaranthus* is a complex taxon with economic importance as well as harmful weeds. We studied the genetic variation and structure of the chloroplast genomes of 22 samples from 17 species of three subgenera. It was found that the length of the chloroplast genome of *Amaranthus* varied from 149,949 bp of *A. polygonoides* to 150,757 bp of *A. albus*. The frequencies of SNPs and InDels in chloroplast genomes were 1.79% and 2.86%, and the variation mainly occurred in the non-coding regions. The longest InDel was 387 bp, which occurred on *ycf2*, followed by 384 bp InDel on *psbM-trnD*. Two InDels in *ndhE-I* on the SSC make the three subgenera clearly distinguished. In LSC, SSC and IRs regions, there were four 30 bp forward and reverse repeats, and the repeats in SSC and LSC were in nearly opposite positions in circular genome structure, and almost divided the circular genome into symmetrical structures. In the topological tree constructed by chloroplast genome, species in subgen. *Amaranthus* and subgen. *Acnida* form monophyletic branches separately and cluster together. *A. albus*, *A. blitoides* and *A. polygonoides* were separated from subgen. *Albersia*, and the rest of subgen. *Albersia* were clustered into a monophyletic branch. The *rpoC2*, *ycf1*, *ndhF-rpl32* were good at distinguishing most amaranths. The *trnk-UUU-atpF*, *trnT-UGU-atpB*, *psbE-clpP*, *rpl14-rps19*, and *ndhF-D* can distinguish several similar species. In general, the chloroplast genome is of certain value for the identification of the similar species of *Amaranthus*, which provides more evidence for clarifying the phylogenetic relationships within the genus.

## Introduction

The genus of *Amaranthus* includes 74 species, of which 55 species native to the Americas and the rest originated from the Euraisa, South Africa and Austrilia/Oceania^1,2^. The genus contains pseudocereals crops such as *A. caudatus* L., *A. cruentus* L., and *A. hypochondriacus* L., leaf vegetables *A. tricolor* and *A. blitum*, endangered plants *A. pumulis*, and agricultural weeds^3^. The Flora of China (eFloras edition) recorded 15 species and two varieties, all of which were alien except for *A. tricolor*^4^. Since then, some authors have successively found new alien amaranths: *A. bouchonii*^5^, *A. tenuifolius*^6^, *A. palmeri*^7^, *A. standelyansus*^8^, *A. powellii*^5^, *A. dubius*^9^ from the collected specimens in China. In port monitoring regions, *A. tuberculatus*, *A. arenicola*, *A. crispus* etc. were newly intercepted and controlled (Xu, unpublished). Among them, *A. arenicola* and *A. crispus* were transient colonization (Xu, unpublished).

**Figure 1:**
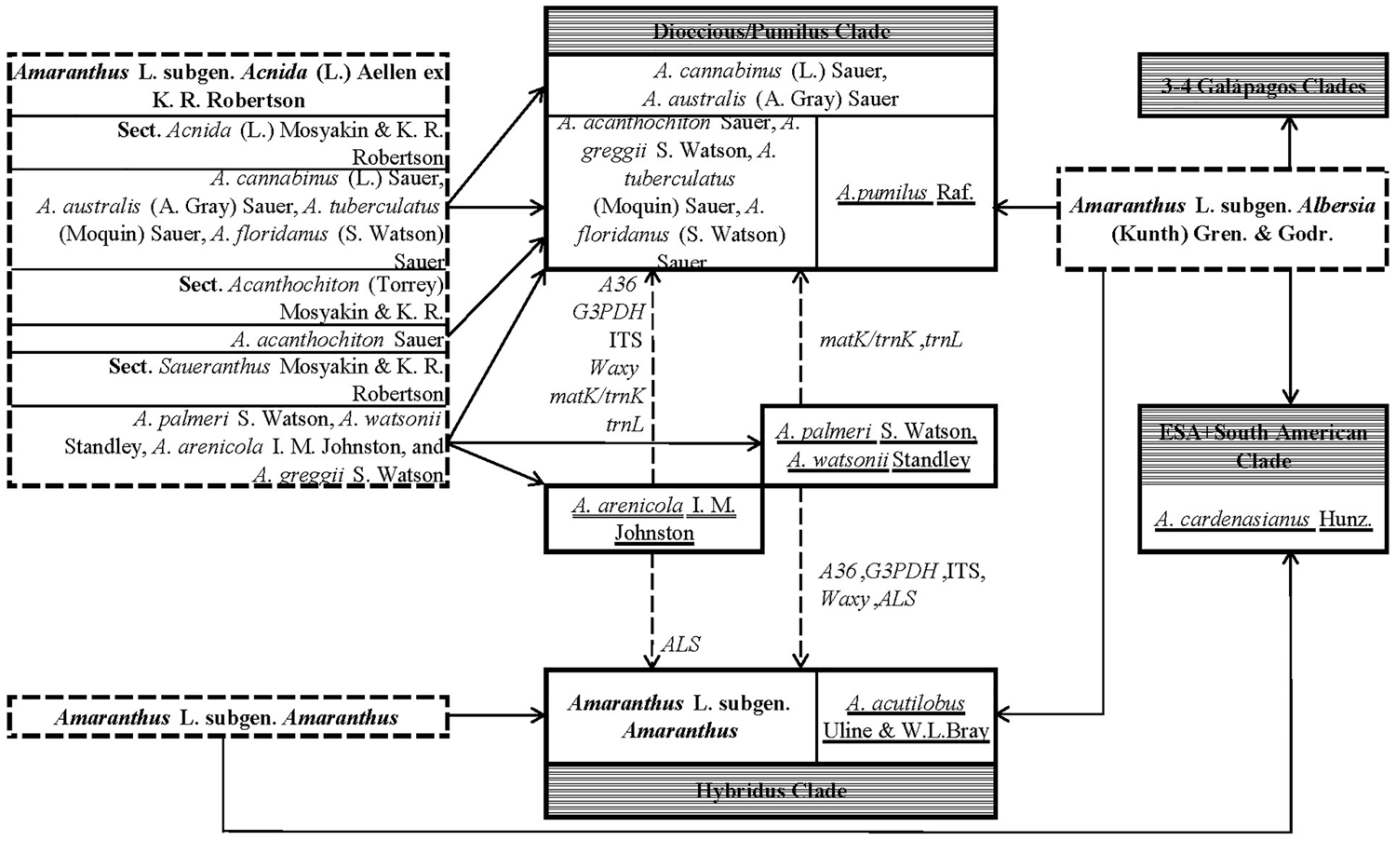
The research status of phylogenetic relationship at the subgenus level in *Amaranthus*. The dotted boxes indicate the traditional three subgenera of *Amaranthus*, the horizontal stripes boxes are the four main clades divided by Waselkov et al. based on nuclear and chloroplast gene sequences. The graphics were drawn using Microsoft Excel of Microsoft Office Professional Plus 2010.

According to inflorescences position, the number of perianth segments and urticle dehiscent/indehiscent^2,10^, as well as dioecious or monoecious, are divided into three subgenera: *Amaranthus* subgen. *Amaranthus*, *Amaranthus* subgen. *Acnida* (L.) Aellen ex K.R.Robertson, and *Amaranthus* subgen. *Albersia* (Kunth) Gren. & Godr.^10,11^ (Fig. 1). Of these, 9 species were listed as “introduced, invasive and noxious plants” in the USDA Plants Database, and 21 species as “agricultural weeds” in the Global Compendium of Weeds^12^. The genus is the focus of weed scientific research^13^, because of these amaranth weeds posed a certain threat to agricultural ecology in the new habitat. *A. palmeri* and *A. tuberculatus* invade gradually into the new continents out of their origins, and were detected their resistant biotypes^14^. Accurate identification of these species is the basis of weed prevention and control. However, the taxonomy of *Amaranthus* has always been difficult, especially because of the large number of complex taxa which are difficult to define due to the interspecific hybridization and gene introgression.

Many authors have studied on the taxonomy and evolution of the genus. The latest taxonomy revision of monoecious species was completed by Bayón^2^, and the comprehensive dioecious taxonomy was Sauer’s monograph^1^. Mosyakin and Robertson gave the most comprehensive classification of the three subgenera under the genus, which is also the most widely used subgenus classification up to now^10,11^ (Fig. 1). Waselkov et al. conducted the phylogenetic analyses of 58 species from three subgenera based on three low-copy nuclear genes and two chloroplast regions, and suggested 4–5 clades of the genus^15^ (Fig. 1). Xu et al. analyzed ITS, *ALS* (domain C, A and D) and *ALS* (domain B and E) and constructed topological trees^14^. In Waselkov et al. studies, both nuclear and chloroplast sequences support the division of *A. pumilus* in subgen. *Albersia* into subgen. *Acnida* or dioecious/Pumilus Clade, the incorporation of *A. acutilobus* into subgen. *Amaranthus* or Hybridus Clade, and the incorporation of *A. cardenasianus* in subgen. *Amaranthus* into ESA + South American Clade of subgen. *Albersia*^15^ (Fig. 1). In ITS, *Waxy* and other sequences, *A. palmeri* and *A. watsonii* of subgen. *Acnida* clustered together with subgen. *Amaranthus*^15^ (Fig. 1). On the chloroplast sequences, *A. palmeri* and *A. watsonii* also returned to subgen. *Acnida*^15^ (Fig. 1). Xu et al. found that *A. arenicola* and subgen. *Amaranthus* formed monophyletic clade on *ALS*^14^, while ITS, *Waxy*, *matK* and other sequences were still clustered on subgen. *Acnida*^15^ (Fig. 1). The subgen. *Albersia* was not a monophyletic group and could be divided into 2–3 clades^15^.

Additionally, chloroplast genome-related studies include: Chaney et al. first reported full chloroplast genomes of *A. hypochondriacus*, *A. cruentus*, *A. caudatus* and their hypothetical wild ancestor species *A. hybridus*, and found 210 single nucleotide polymorphisms (SNPs) and 122 insertion/deletion polymorphisms (InDels) compared to the reference chloroplast genome^16^. Viljoen et al. studied chloroplast genomes and *matK*, *rbcL*, ITS in 59 accessions of 9 species of subgen. *Amaranthus* and 4 species of subgen. *Albersia*, and mainly focused on the genetic relationship between wild and domesticated grain amaranths^17^. At present, studies on the chloroplast genome of *Amaranthus* are mainly focused on the grain amaranths, and there is a lack of overall studies on the three subgenera.

In this paper, combined with the problems existing in the classification and evolution of the genus, and the research need of the genetic variation of alien invasive amaranths, we collected alien species of 3 subgenera, and perform complete analysis of the chloroplast genome, in order to further understand the feature of the chloroplast genomes of *Amaranthus*, and the critical regions of chloroplast genomes used to explain the phylogenetic relationship of the genus, especially the evolution of *A. palmeri* and *A. spinosus*, *A. tuberculatus* and *A. arenicola*, *A. spinosus* and *A. dubius*. The results will provide a new basis for the taxonomic revision, phylogenetic evolutionary, weed evolutionary biology and the development of genetic resources.

## Results

### Genomic features

The quadripartite structure of 22 samples of 17 species in *Amaranthus* consists of a large single-copy region (LSC with 83, 382–84, 062 bp), a small single-copy region (SSC with 17, 937 – 18, 124 bp), and a pair of inverted repeat regions (IRs with 23, 964–24, 357 bp). The full length of the 22 cp genomes ranges from 149,949 bp in *A. polygonoides* to 150, 756 bp in *A. albus* (Table 1). The chloroplast genome sequences were deposited in GenBank (Table 1).

**Table 1:** Summary information for the chloroplast genomes of *Amaranthus.*

No	Species	Comparison of genome length (bp)				GC content (%)	Genbank accession
		Total	LSC	SSC	IRs		
1	*Amaranthus retroflexus*	150,244	83,605	17,937	24,351	36.6	MN091971
2	*Amaranthus dubius*	150,524	83,880	17,940	24,352	36.6	MN091972
3	*Amaranthus spinosus* 113	150,523	83,879	17,940	24,352	36.6	MT526784
4	*Amaranthus spinosus* 11,902	150,524	83,880	17,940	24,352	36.6	MT526783
5	*Amaranthus hypochondriacus	150,523	83,878	17,941	24,352	36.6	*MG836505
6	*Amaranthus hybridus*	150,690	84,062	17,948	24,340	36.6	MT559305
7	*Amaranthus palmeri*	150,731	84,010	18,027	24,347	36.6	MN091990
8	*Amaranthus arenicola* JSTZ	150,632	83,901	18,039	24,346	36.6	MN091969
9	*Amaranthus arenicola* HBTS	150,630	83,899	18,039	24,346	36.6	MZ152791
10	*Amaranthus tuberculatus* GZW	150,679	83,945	18,042	24,346	36.6	MT559304
11	*Amaranthus tuberculatus* 11,994	150,695	83,961	18,042	24,346	36.6	MN091967
12	*Amaranthus tuberculatus* 12,194	150,696	83,962	18,042	24,346	36.6	MN091968
13	*Amaranthus blitum*	150,621	83,806	18,057	24,379	36.6	MT526777
14	*Amaranthus crispus*	150,567	83,793	18,060	24,357	36.6	MT526778
15	*Amaranthus standleyanus* 11,960	150,567	83,793	18,060	24,357	36.6	MT526781
16	*Amaranthus standleyanus* 7433	150,568	83,794	18,060	24,357	36.6	MT526782
17	*Amaranthus tunetanus*	150,581	83,805	18,062	24,357	36.6	MT526780
18	*Amaranthus deflexus*	150,256	83,489	18,065	24,351	36.6	MT526776
19	*Amaranthus capensis*	150,707	83,928	18,075	24,352	36.6	MT526779
20	*Amaranthus blitoides*	150,667	83,878	18,089	24,350	36.5	MT526786
21	*Amaranthus albus*	150,756	83,943	18,111	24,351	36.5	MT526785
22	*Amaranthus polygonoides*	149,948	83,896	18,124	23,964	36.5	MT472619

*Chloroplast genomic data for *Amaranthus hypochondriacus* were obtained from GenBank.

The total GC content was 36.5% to 36.6%, only *A. albus*, *A. blitoides* and *A. polygonoides* have a GC content of 36.5% (Table 1). The chloroplast genome contains a total of 133 genes, including 88 protein-coding genes, 37 tRNA genes, and 8 rRNA genes, 18 of which were duplicated in the inverted repeat regions (see Supplementary Table S1 online). The gene *rps12* was trans-spliced; the 50-end exon was located in the LSC region, whereas the 30- intron and exon were duplicated and located in the inverted repeat regions. The partial duplicate of *rps19* and *ycf1* genes appeared as pseudogenes as they lost their protein-coding ability. 16 genes have introns.

### Variants of cp genomes

The length of the SSC region was conserved among the subgenera by comparing the length of the chloroplast genomes of 22 individuals from 17 species. *A. palmeri*, *A. tuberculatus* and *A. arenicola* in subgen. *Acnida* were 18,027–18,042 bp in length, the SSC length of 5 species of subgen. *Amaranthus* was 17,937–17,948 bp, and the SSC length of 8 species of subgen. *Albersia* was 18,057–18,124 bp (Tables 1, 2). There were about 77 bp InDels in *ndhE-G* and 180 bp InDels in *ndhG-I*, which induced the variation of SSC length among subgenera (Table 2; see Supplementary Fig. S1 online). The frequencies of SNPs and InDels in the chloroplast genomes of the 17 species were 1.79% and 2.86%, respectively (Table 3). The frequencies of SNPs and InDels in the genes were 1.22% and 1.14%, and the frequencies of SNPs and InDels in the intergenic spacer were 3.25% and 7.32%, respectively (Table 3). In general, the variation mainly occurred in the intergenic spacer region, and InDels mainly occurred in the non-coding region (Table 3). The longest InDel was 387 bp, which occurred on *ycf2*, followed by 384 bp InDel on *psbM-trnD*.

**Table 2:** Length differences of chloroplast genome SSC regions at the subgenus level in *Amaranthus.*

Average ± standard deviation (bp)	subgen. *Amaranthus*	subgen. *Albersia*	subgen. *Acnida*
subgen. *Amaranthus*	17,941.00 ± 3.37	–	–
subgen. *Albersia*	18,025.56 ± 67.94	18,076.30 ± 22.68	–
subgen. *Acnida*	17,989.75 ± 48.95	18,062.13 ± 25.83	18,038.50 ± 5.32

**Table 3:** Variation of the chloroplast genomes in *Amaranthus.*

Region	Length (bp)	SNPs		InDels	
		Numbers	Frequency (%)	Numbers	Frequency (%)
Consensus sequence	152,519	2735	0.0179	4363	0.0286
Gene	110,128	1354	0.0123	1258	0.0114
CDS	80,201	1034	0.0129	862	0.0107
tRNA	2780	9	0.0032	0	0
rRNA	9042	6	0.0007	0	0
Intron	18,105	305	0.0168	396	0.0219
IGS	42,391	1381	0.0326	3105	0.0732

### Repeat and SSR analyses

**Figure 2:**
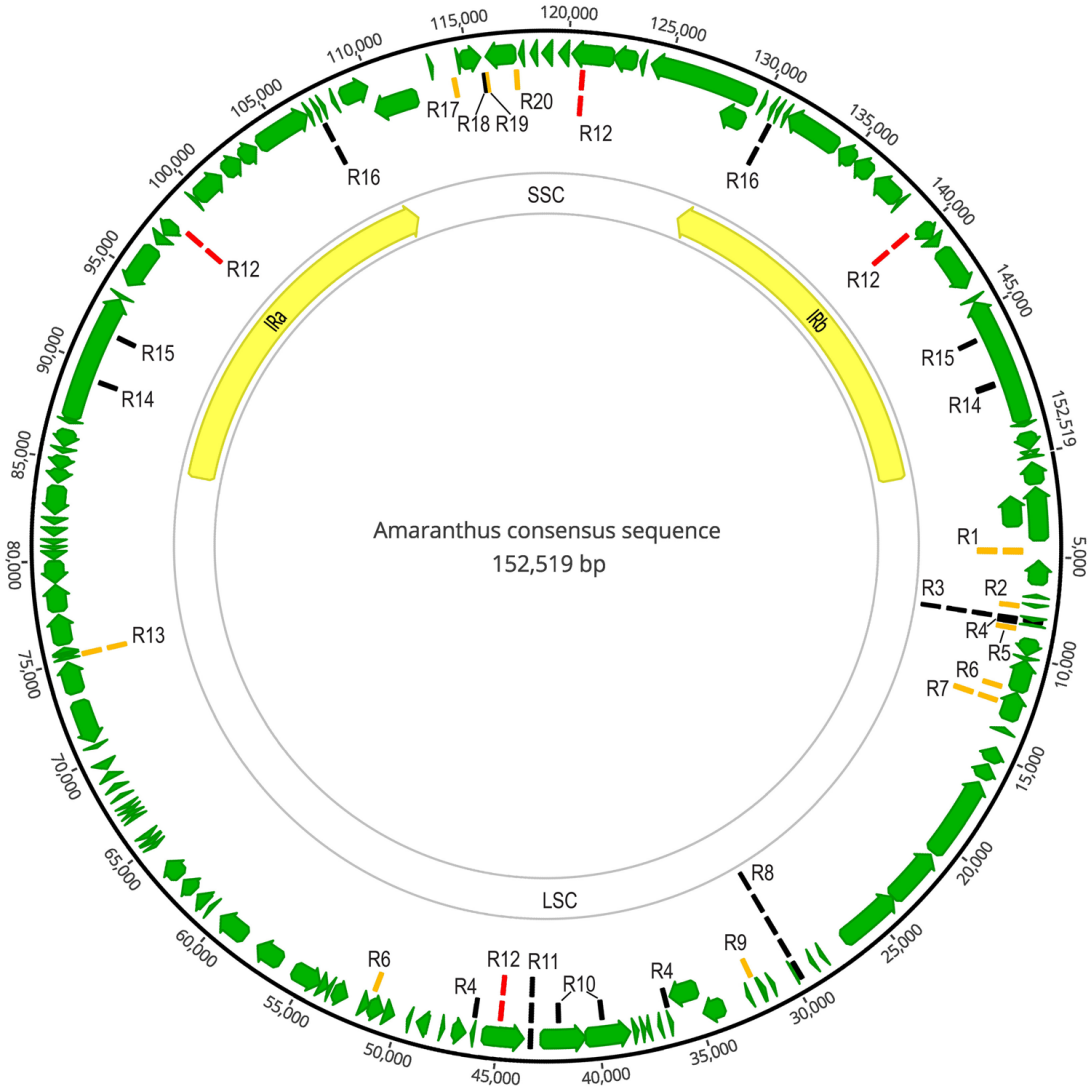
The distribution of repeat sequences at 30 loci in *Amaranthus*. “R” is short for repeat. The red line segment R12 and the black line segments are repeats in all 17 species, the orange line segment represents a repeating sequence in some species. A repeat with only one line segment indicates that there is only one repeat at the site, and vice versa indicates that there are several different repeats at the site. The chloroplast genome figure was generated by the Geneious Prime v. 2020.1.2 software.

Each species has 28 to 38 repeats, distributed in 30 locations, including 11 to 14 forward repeats, 11 to 17 palindromic repeats, and 6 to 8 reverse repeats ranging from 30 to 64 bp in length. There were 19 common repeats locations, of which 11 had no variation and 8 had variation in length. The R3, R8, R11 and R13 had the most abundant variation (Fig. 2). The R12 (forward and reverse repeats) was distributed in LSC, IRa, SSC and IRb. The R12 on SSC is almost opposite to R12 on LSC, dividing the entire circular genome into two parts of nearly equal length. The repeats on LSC were mainly concentrated near Repeat 12 (loci 29,572–46,282), loci 8166–8327, loci 29,572 and loci 75,230. The repeats on IRs are constant within the genus. There were two common repeats in SSC, and one was a palindrome sequence shared by subgen. *Acnida*, subgen. *Amaranthus*, and *A. albus*.

MISA analysis showed that each cp genome of *Amaranthu*s contained 29–39 SSRs (see Supplementary Table S4 online). On average, the number of SSR types from more to less was mono-, tetra-, di-, tri-, penta- and hexa-nucleotides in order (see Supplementary Table S4 online). About 55.56% of those SSRs were composed of A or T bases. Among all SSRs, most loci located in LSC (77.78%) and IGS (71.91%). About 12 repeat motifs were shared by all species in the genus while the remaining motifs were species-specific or subgenus-specific (see Supplementary Table S4 online). Different combinations of SSR markers could distinguish all species except *A. standleynaus* and *A. crispus*, *A. dubius* and *A. spinosus* (see Supplementary Table S4 online).

### Phylogenetic trees of whole chloroplast genomes

**Figure 3:**
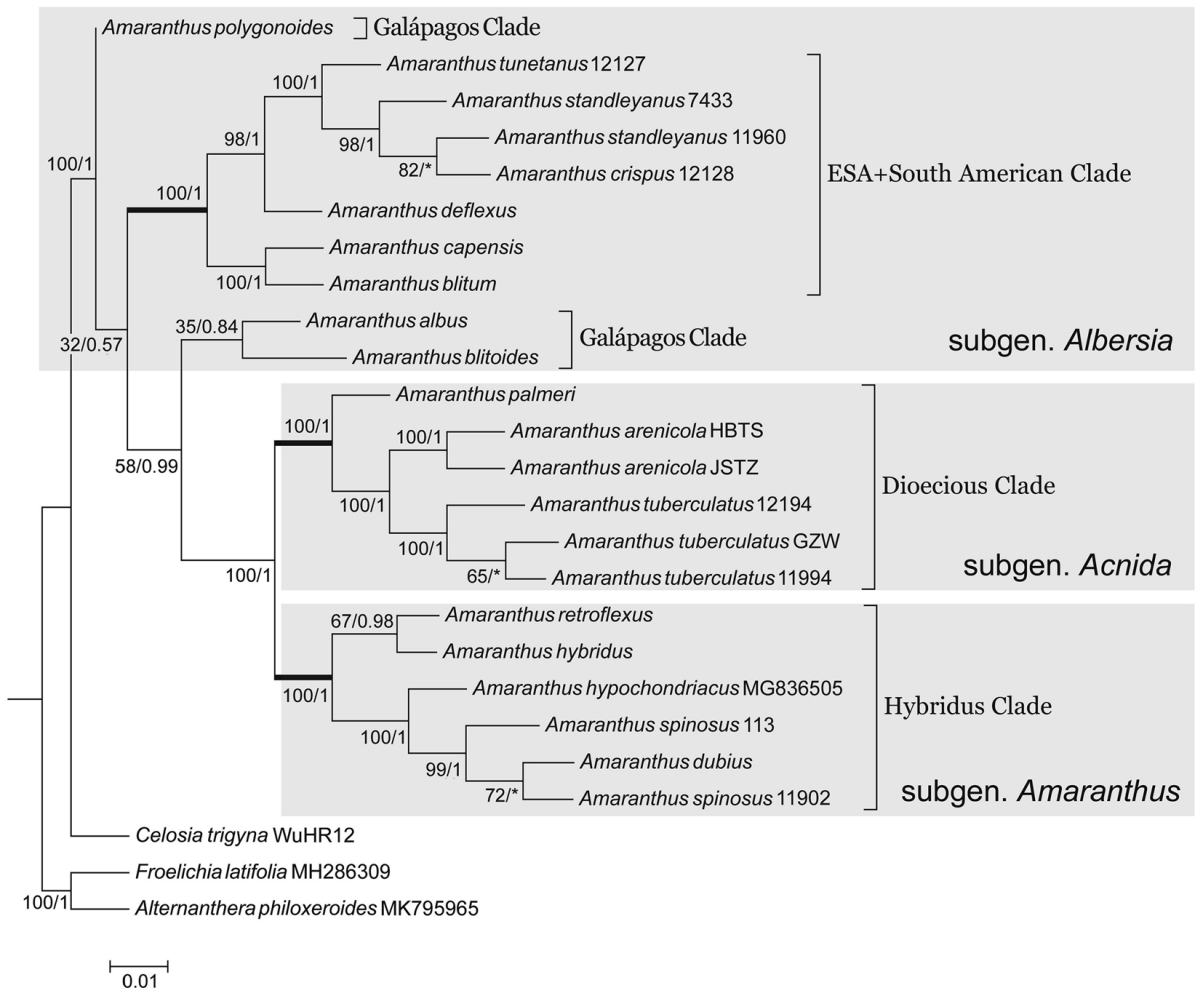
A maximum likelihood topological tree based on chloroplast genome of *Amaranthus* and three outgroups. Values at each node indicate maximum likelihood bootstrap support (BS)/Bayesian inference posterior probability (PP) value. Individuals marked with grey backgrounds represent major monophyletic branches in the genus. The newick format files are imported into MEGA version 6 to generate the final topology tree.

The topologies of the phylogenetic trees constructed by maximum likelihood and Bayesian methods were the same basically. *A. palmeri*, *A. arenicola* and *A. tuberculatus* clustered together (BS/PP = 100/1) to form subgen. *Acnida*, or the Dioecious Clade (Fig. 3). *A. hybridus*, *A. hypochondriacus*, *A. dubius*, *A. spinosus*, *A. retroflexus* clustered together (BS/PP = 100/1) to represent subgen. *Amaranthus*, or the Hyridus Clade (Fig. 3). And the above two clades were very close (BS/PP = 100/1) (Fig. 3). *A. albus* and *A. blitoides* were clustered with low/moderate value (BS/PP = 35/0.84) and separated from subgen. *Albersia* and were closely related to subgen. *Amaranthus* and subgen. *Acnida* (BS/PP = 58/0.99) (Fig. 3). Among the three species of subgen. *Albersia* distributed in Galápagos, *A. polygonoides* became a single basal branch. The other two species, *A. albus* and *A. blitoides*, formed a separate clade (Galápagos Clade). The rest of subgen. *Albersia* were clustered into one branch, namely the ESA + South American Clade (BS/PP = 100/1) (Fig. 3).

### Hotspots for *Amaranthus*

**Figure 4:**
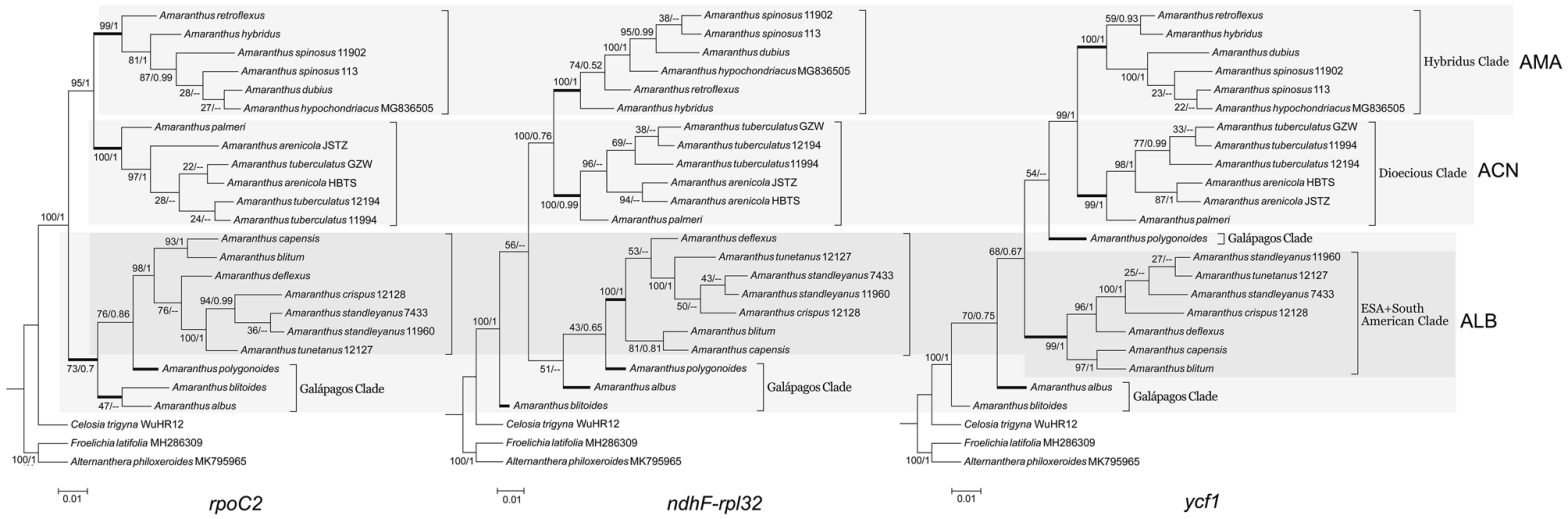
Three maximum likelihood topological trees based on *rpoC2*, *ndhF-rpl32* and *ycf1* of *Amaranthus* and three outgroups. Values at each node indicate maximum likelihood bootstrap support (BS)/Bayesian inference posterior probability (PP) value. “AMA” represented the subgen. *Amaranthus*, “ACN” represented the subgen. *Acnida*, “ALB” represented the subgen. *Albersia*. The newick format files are imported into MEGA version 6 to generate the final topology tree.

The partially qualified fragment regions searched by exhaustive method were overlapped, and the overlapped regions were combined together as a hotspot region. Finally, 16 hotspot fragments with a length of 737 to 2818 bp were obtained, and the SNP variation frequency ranged from 0.78 to 1.49% (see Supplementary Table S3 online). The topological trees constructed by the alignments of these 17 hot fragments and the topological trees constructed by the alignment sequences of each gene and intergenic spacer were consistent with the chloroplast genome topological tree, namely, the hotspots with more than 90% bootstrap value support for the subgen. *Amaranthus*, subgen. *Acnida* and subgen. *Albersia* branch (excluding *A. albus*, *A. polygonoides*, and *A. blitoides*) were *ndhF-rpl32*, *ycf1* and *rpoC2* (Fig. 4).

In several similar taxa, there were 25 InDels and 11 SNPs between *A. tunetanus* and *A. standleyanus*. *A. crispus* and *A. standleyanus* had no difference. There are 46 SNPs and 144 InDels between *A. arenicola* and *A. tuberculatus*. By sequence alignment and variation analysis, it was found that *trnK-UUU*-*atpF*, *trnT-UGU*-*atpB*, *psbE*-*clpP*, *rpl14*-*rps19*, *ndhF-D* could be used to distinguish *A. tunetanus* from *A. standleyanus*, *A. crispus*, and *A. arenicola* from *A. tuberculatus*.

## Discussion and conclusion

The results obtained in this study in limited samples were basically consistent with previous studies based on chloroplast gene sequences.

In this study, the SSC length of *A. hybridus* and other species in subgen. *Amaranthus* was basically consistent with previous studies on four grain amaranths^16^. Chaney et al. reported that the chloroplast genome of *Amaranthus* contained 111 genes, while Viljoen et al. reported that *A. tricolor* contained 140 genes^16,17^. Data in both studies showed minor errors and duplications. After repeated data proofreading in this study, 133 genes were confirmed in the chloroplast genome of *Amaranthus*. In addition, due to the inclusion of more amaranths than the former sample, the number of loci polymorphisms found increased to 2735 SNPs and 4363 InDels.

In addition, Chaney et al. found 29–37 SSRs in four grain amaranths^16^. In our study, 29 to 39 SSRs were identified. After statistical analysis and labeling of the SSRs from each sample, different combinations of SSR markers were found to be able to distinguish the similar species: *A. arenicola* and *A. tuberculatus*, *A. standleyanus* and *A. tunetanus*. In terms of repeats, Chaney et al. reported 34 to 37 repeats, including 14 to 16 forward repeats and 20 to 21 palindromes^16^. This study found 28 to 38 repeats, 11 to 14 forward repeats, 11 to 17 palindromic repeats, and 6 to 8 reverse repeats. Moreover, the distribution of repeats on the chloroplast genome of *Amaranthus* is found to be regular, such as the distribution of R12. This symmetrical structure should play an important role in the recombination or stabilization of *Amaranthus* chloroplast genes.

The topological tree constructed from the chloroplast genome is basically consistent with the phylogenetic results of Waselkov et al. using the chloroplast sequence of *matK/trnK-UUU* and *trnL-UAA*^15^. Namely, *A. albus*, *A. blitoides*, *A. polygonoides* from subgen. *Albersia* points out to become part of the Galápagos Clades. *A. palmeri* and *A. spinosus* belong to the original dioecious subgenus. *A. albus*, *A. blitoides* and *A. polygonoides* were clustered in subgen. *Albersia* with moderate or high bootstrap value by using a few genes or sequences of ITS, *ALS* and *rpoC2* or incomplete phylogenetic relationships of *Amaranthus*^14,15^. As the number of species and gene sequences increased, *A. albus*, *A. blitoides*, and *A. polygonoides* apparently diverged from subgen. *Albersia* into separate clades^15^. This study also supports the conclusion that the original subgen. *Albersia* is not a natural taxonomic group.

The chloroplast capture event speculated to occur in Waselkov et al. was further confirmed. In combination with nuclear gene studies, the relationship between *A. palmeri* and *A. spinosus*^15,18^, and *A. palmeri* is one of the few species in the dioecious subgenera (*A. watsonii* and *A. arenicola*) that have the characteristics of five perianth segments, suggestting that the hybridization of a species of subgen. *Acnida* and *A. spinosus* in the earlier stage may have led to the chloroplast capture event, which eventually resulted in the formation of *A. palmeri.*

In combination with previous studies, we found that *rpoC2*, *ycf1* and *ndhF-rpl32* sequences can be used for phylogenetic and taxonomic identification of *Amaranthus*, according to the principle of similar topological tree branches with the whole chloroplast genome (Fig. 4). However, these three sequences cannot effectively distinguish the similar species. In previous studies on the ITS and chloroplast genes *matK/trnK-UUU* and *trnL-UAA* of *A. arenicola* and *A. tuberculatus*, the two species were almost indistinguishable^14,15^. In this study, it was found that there was only one SNP site difference in *matK/trnK-UUU* between *A. arenicola* and *A. tuberculatus*, while their ITS^14,19^ and *trnL-UAA* sequences showed no difference^19^. In contrast, there are 46 SNPs and 144 InDels between *A. arenicola* and *A. tuberculatus* on chloroplast genomes. The ITS sequences of *A. crispus* and *A. tunetanus* were the same, with only one base difference from *A. standleyanus*^14^. However, there were 25 InDels and 11 SNPs in the chloroplast genomes of *A. tunetanus* and *A. standleyanus*. The five newly discovered regions, *trnK-UUU-atpF*, *trnT-UGU-atpB*, *psbE-clpP*, *rpl14-rps19*, and *ndhF-D*, have enough parsimony information sites to distinguish several similar species.

In conclusion, the chloroplast genome is of some significance to the phylogenetic study of *Amaranthus*. However, the study of interspecific and intraspecific gene variation had better be combined with the morphological characteristics of the samples. For species whose morphology is difficult to define, identification errors often occur in samples, and thus the results of molecular analysis are correspondingly wrong. Additionally, the inconsistency of phylogenetic relationships between the chloroplast genome and the nuclear gene sequence of *Amaranthus* may provide new evidence for the evolution and origin of some species.

## Materials and methods

### Plant samples, DNA extraction, and sequencing

In this experiment, 21 samples from 16 species of *Amaranthus* and three species as outgroups were used for chloroplast genome analysis (see Supplementary Table S4 online). All samples were collected from the wild population around the processing plants, wastelands, wharfs in the port supervision area except *A. deflexus*. *A. deflexus* is a common weed collected from the wasteland near the Spanish fields. The specimen collection team was composed of officials of the National Port Weed Monitoring Office of CIQ. The samples collected were approved by the customs and other plant quarantine authorities, and complied with *Biosafety Law of the People's Republic of China* (Order of the President of the People's Republic of China, No.56) and ISPM 09: *Guidelines for pest eradication programmes* adopted by International Plant Protection Convention. Habitat and biodiversity were not damaged, and endangered species were not involved. The specimens were deposited at the plant inspection and quarantine institute of Chinese Academy of Inspection and Quarantine (CAIQ) (Beijing, China). All samples were mature plants with flowers and fruits, and identified according to the classification monographs of *Amaranthus* by Sauer^20^, Mosyakin and Robertson^3^, and Bayón^2^. Total genomic DNA was extracted from the silica-dried leaf tissues using Plant Genomic DNA Kit (Tiangen Biotech Co., China). Genomic DNA of each individual was indexed by a barcode and then pooled together with other samples for sequencing in one lane of HiSeq 2500 (Illumina) (Novogene, Beijing, China).

### Genome assembly and annotation

The paired-end sequencing data (2 × 150 bp) were used to assemble its complete chloroplast genome. Sequencing adapters and barcodes were trimmed and low quality reads with Q value ≤ 30 removed. Trimmed paired end reads were mapped to the chloroplast sequence of *A. hypochondriacus* (GenBank accession: MG 836,505), with default parameters. The reads were assembled using the Geneious Prime v. 2020.1.2 (Biomatters, Auckland, New Zealand). The consensus chloroplast sequence of *Amaranthus* spp. was retrieved separately and used as a reference for several rounds of mapping of itself reads in order to validate its consensus chloroplast sequence. All trimmed and quality-filtered sequence reads have been deposited in Genbank of NCBI. Non-mapped reads, which are assumed to be of non-plastid origin, were excluded from further analysis. The complete chloroplast genome sequence was annotated using the Geneious Prime v. 2020.1.2 (Biomatters, Auckland, New Zealand) by comparing with the genome of *A. hypochondriacus* (GenBank accession: MG 836505). The assembled and annotated *Amaranthus* spp. chloroplast genome sequence was deposited at NCBI (Table 1), the alignment used for constructing the tree in Fig. 3 was deposited at NCBI Sequence Read Archive (SRA) (Submission ID: SUB11230935, BioProject ID: PRJNA820520).

### Genome comparative analysis

A comparative plot of full alignment with annotations of the 22 chloroplast genomes was produced and the nucleotide variability was calculated by Geneious prime v. 2020.1.2 (Biomatters, Auckland, New Zealand) to analyze the total number of mutations. The comparative analysis included the reference sequence *A. hypochondriacus.* The alignment has released in a public database.

### Characterization of repeat sequences and SSRs

We used REPuter^21^ to identify the position and size of repeat sequences, which included forward, palindromic, reverse, and complement repeats in the chloroplast genomes of *Amaranthus*. The sequence identity and minimum length of repeat size was set to > 90% and 30 bp. MISA perl script was used to detect the simple sequence repeats (SSRs) in the chloroplast genomes^22^. The thresholds for mono-, di-, tri-, tetra-, penta-, and hexa-nucleotide SSRs were 10, 5, 4, 3, 3, and 3 repeat units, respectively.

### Phylogenetic trees

All phylogenetic analyses were undertaken by the Geneious Prime v. 2020.1.2 software (Biomatters, Auckland, New Zealand), based on the chloroplast genomes of 25 sequences of 20 species (see Supplementary Table S4 online), including the reference chloroplast genome *A. hypochondriacus*, and three outgroups, *Celosia trigyna* (Genbank Accession: MN057637), *Alternanthera philoxeroides* (Genbank Accession: MK795965) and *Froelichia latifolia* (Genbank Accession: MH286309). The 25 chloroplast genome sequences were aligned using MAFFT^23^. The DNA substitution model (GTR + I + G model) was chosen using jModelTest 2.1.6^24^, and used in maximum likelihood (ML) analysis and Bayesian inference. ML analysis was conducted using RAxML version 8.2.11^25^ on the Geneious Prime v. 2020.1.2 (Biomatters, Auckland, New Zealand). Bayesian inference was conducted using MrBayes 3.2.6^26^ with Ngen = 1 000 000, Samplefreq = 200, and Burninfrac = 0.25. The newick format files are imported into MEGA version 6 to generate the final topology tree^27^.

### Search for hotspots

Two methods were used to select suitable regions: (1) search with SNP sites greater than 10 per 1000 bp based on exhaustive method by Microsoft Excel 2010; (2) the gene and gene spacer were analyzed one by one manually. Finally, a topological tree was constructed for the searched region and compared with the chloroplast genome topological tree to test the resolution authenticity of this region. The topology tree construction method is consistent with the method in 2.6.

### Collection statement

The samples collected were approved by the customs and other plant quarantine authorities, and complied with *Biosafety Law of the People's Republic of China* (Order of the President of the People's Republic of China, No.56). Habitat and biodiversity were not damaged, and endangered species were not involved.

## Supplementary Information

Supplementary Information.

## Data Availability

The data generated and analyzed in this study are available from the authors on request. The alignment has been submitted in a public dataset.
