# Supplementary material for: Genetic variation and structure of complete chloroplast genome in alien monoecious and dioecious Amaranthus weeds
Source: Sci Rep. 2022 May 18;12:8255. doi: 10.1038/s41598-022-11983-2 (PMC9117656; doi:10.1038/s41598-022-11983-2)
Supplement: Supplementary file 1 — Supplementary Information. [file 41598_2022_11983_MOESM1_ESM.docx]

Genetic variation and structure of complete chloroplast genome in alien monoecious and dioecious *Amaranthus* weeds

Han Xu^1*^, Ning Xiang^1^, Wei Du^2^, Junhua Zhang^1^, Yongjiang Zhang^1*^

^1^Institute of Plant Inspection and Quarantine, Chinese Academy of Inspection and Quarantine, Beijing 100176, China

^2^Agricultural Technology Extension Station of Ningxia, Yinchuan 750001, China

^*^To whom correspondence should be addressed. E-mail: xuhangin@gmail.com, [zhangyjpvi@yeah.net](mailto:zhangyjpvi@yeah.net)

**Supplementary Information**

**Table S1** Genes contained in the sequenced amaranths chloroplast genomes.

**Table S2** Distribution of SSRs in the chloroplast genome of *Amaranthus*.

**Table S3** The hotspot regions obtained based on the exhaustive method.

**Table S4** Materials information of *Amaranthus*.

**Figure S1** The main insertion and deletion regions on SSC of *Amaranthus* were *ndhE-G* (77 bp) and *ndhG-I* (180 bp). “AMA” represented the subgen. *Amaranthus*, “ACD” represented the subgen. *Acnida*, “ALB” represented the subgen. *Albersia*.

**Table S1** Genes contained in the sequenced amaranths chloroplast genomes.

| **Category** | | **Gene group** | **Gene names** |
| --- | --- | --- | --- |
| Protein-coding genes | Photosynthesis | Subunits of ATP synthase | *atpA, atpB, atpE, atpF^a^, atpH, atpI* |
|  |  | Subunits of NADH-dehydrogenase | *ndhA^a^, ndhB^a^ (×2), ndhC, ndhD, ndhE, ndhF, ndhG, ndhH, ndhI, ndhJ, ndhK* |
|  |  | Subunits of cytochrome b/f complex | *petA, petB, petD, petG, petL, petN* |
|  |  | Subunits of photosystem I | *psaA, psaB, psaC, psaI, psaJ* |
|  |  | Subunits of photosystem II | *psbA, psbB, psbC, psbD, psbE, psbF, psbH, psbI, psbJ, psbK, psbL, psbM, psbN, psbT, psbZ* |
|  |  | Subunit of RuBisCO | *rbcL* |
|  | Other genes | Subunit of acetyl-CoA-carboxylase | *accD* |
|  |  | c-type cytochrome synthesis gene | *ccsA* |
|  |  | Envelope membrane protein | *cemA* |
|  |  | Protease | *clpP^a^* |
|  |  | Translational initiation factor | *infA* |
|  |  | Maturase | *matK* |
|  | Unknown function | Conserved open reading frames | *ycf1 (×3), ycf2 (×2), ycf3, ycf4* |
|  | Self-replication | Large subunit of ribosome | *rpl2 (×2), rpl14, rpl16b, rp20, rpl22, rpl32, rpl33, rpl36* |
|  |  | DNA-dependent RNA polymerase | *rpoA, rpoB, rpoC1^a^, rpoC2* |
|  |  | Small subunit of ribosome | *rps2, rps3, rps4, rps7 (×2), rsp8, rps11, rps12^a^ (×3), rps14, rps15, rps16^a^, rps18, rps19 (×2)* |
| rRNA genes |  | rRNA genes | *rrn4.5S (×2), rrn5S (×2), rrn16S (×2), rrn23S (×2)* |
| tRNA genes |  | tRNA genes | *trnA-UGC^a^ (×2), trnC-GCA, trnD-GUC, trnE-UUC, trnF-GAA, trnfM-CAU, trnG-UCC^a^, trnG-GCC, trnH-GUG, trnI-CAU (×2), trnI-GAU^a^ (×2), trnK-UUU^a^, trnL-CAA (×2), trnL-UAA^a^, trnL-UAG, trnM-CAU, trnN-GUU (×2), trnP-UGG, trnQ-UUG, trnR-ACG (×2), trnR-UCU, trnS-GGA, trnS-UGA, trnS-GCU, trnT-GGU, trnT-UGU, trnV-GAC (×2), trnV-UAC^a^, trnW-CCA, trnY-GUA* |

**Table S2** Distribution of SSRs in the chloroplast genome of *Amaranthus*.

| **Region** | | | **Locus** | **SSR type** | **pol** | **alb** | **blo** | **blu** | **cap** | **def** | **cri** | **sta 2** | **sta 7433** | **sta 11960** | **ret** | **hyb** | **hyp** | **dub** | **spi** | **spi 11902** | **pal** | **are** | **are** | **tub 11994** | **tub 12194** | **tub GZW** |
| --- | --- | --- | --- | --- | --- | --- | --- | --- | --- | --- | --- | --- | --- | --- | --- | --- | --- | --- | --- | --- | --- | --- | --- | --- | --- | --- |
| LSC | IGS | *trnK-UUU-rps16* | 4457-4468 | (AAGA)3 |  |  | * |  |  |  |  |  |  |  |  |  |  |  |  |  |  |  |  |  |  |  |
|  |  |  | 4719-4730 | (AT)6 |  | * |  | * | * | * | * | * | * | * |  |  |  |  |  |  |  |  |  |  |  |  |
|  |  |  | 4743-4806 | (ATT)4 |  | * | * |  |  |  |  |  |  |  |  |  |  |  |  |  | * | * | * | * | * | * |
|  |  |  | 4762-4782 | (AT)8 |  |  | * |  |  |  |  |  |  |  |  |  |  | * | * | * |  |  |  |  |  |  |
|  |  |  | 4782-4793 | (TTA)4 |  |  |  |  |  |  |  |  |  |  |  |  | * | * | * | * |  |  |  |  |  |  |
|  |  |  | 4795-4806 | (ATT)4 | * | * | * |  |  |  |  |  |  |  |  |  |  |  |  |  |  |  |  |  |  |  |
|  |  |  | 4796-4807 | (TTA)4 |  |  |  |  |  |  |  |  |  |  | * | * | * | * | * | * |  |  |  |  |  |  |
|  |  |  | 4878-4892 | (TAT)5 |  |  |  |  |  |  |  |  |  |  |  | * |  |  |  |  |  |  |  |  |  |  |
|  |  |  | 4880-4894 | (TTA)4,5 |  |  | * | * |  | * | * | * | * | * | * |  | * | * | * | * |  |  |  |  |  |  |
|  |  |  | 4882-4911 | (ATT)5 |  |  |  |  |  |  |  |  |  |  |  |  |  |  |  |  | * | * | * | * | * | * |
|  |  |  | 4913-4924 | (TTA)4 |  |  |  |  |  |  |  |  |  |  | * |  |  |  |  |  | * | * | * | * | * | * |
|  |  |  | 4929-4943 | (TAT)5 |  |  |  |  |  |  |  |  |  |  |  | * |  |  |  |  |  |  |  |  |  |  |
|  | Intron | *rps16* | 5798-5809 | (T)12 |  |  |  |  | * |  |  |  |  |  |  |  | * | * | * | * |  |  |  |  |  |  |
|  | IGS | *rps16-trnQ-UUG* | 6789-6805 | (A)13 |  |  |  |  |  |  | * | * | * | * |  |  |  |  |  |  |  |  |  |  |  |  |
|  | IGS | *trnQ-UUG-psbK* | 7257-7269 | (T)12 |  | * |  |  |  |  |  |  |  |  |  |  |  |  |  |  |  |  |  |  |  |  |
|  | IGS | *psbK-psbI* | 7851-7863 | (T)12,13 |  |  | (T) |  | (T) | (T) |  |  |  |  |  |  |  | (T) | (T) | (T) | (T) | (T) | (T) | (T) | (T) | (T)12 |
|  |  |  |  |  |  |  | 12 |  | 13 | 12 |  |  |  |  |  |  |  | 13 | 13 | 13 | 12 | 12 | 12 | 12 | 12 |  |
|  | IGS | *psbI-trnS-GCU* | 8172-8184 | (T)12,13 |  |  |  |  | (T) |  | (T) |  | (T) | (T) |  |  |  |  |  |  |  |  |  | (T) | (T) | (T)13 |
|  |  |  |  |  |  |  |  |  | 12 |  | 12 |  | 12 | 12 |  |  |  |  |  |  |  |  |  | 13 | 13 |  |
|  | tRNA | *trnS-GCU* | 8214-8226 | (A)12 |  |  |  |  | * |  |  |  |  |  | * |  |  | * | * | * |  |  |  |  |  |  |
|  | IGS | *trnS-GCU-trnG-UCC* | 8654-8666 | (A)12,13 |  | (A)13 | (A)13 |  |  | (A)12 |  |  |  |  |  |  |  |  |  |  |  |  |  |  |  |  |
|  | Intron | *trnG-UCC* | 9466-9484 | (T)12 |  |  |  |  | * |  |  |  |  |  |  |  |  |  |  |  |  |  |  |  |  |  |
|  | IGS | *atpA-atpF* | 11720-11734 | (T)13,14,15 | (T) | (T) | (T) | (T) | (T) | (T) | (T) | (T) | (T) | (T) |  |  |  |  |  |  |  |  |  |  |  |  |
|  |  |  |  |  | 14 | 13 | 15 | 15 | 15 | 13 | 13 | 13 | 14 | 13 |  |  |  |  |  |  |  |  |  |  |  |  |
|  | IGS | *atpF-atpH* | 13305-13319 | (TTTAT)3 |  |  |  |  |  |  |  |  |  |  |  | * |  |  |  |  |  |  |  |  |  |  |
|  |  |  | 13459-13470 | (GGAA)3 | * | * | * | * | * | * | * | * | * | * | * | * | * | * | * | * | * | * | * | * | * | * |
|  | CDS | *rpoC2* | 16683-16696 | (T)14 |  |  |  | * |  |  |  |  |  |  |  |  |  |  |  |  |  |  |  |  |  |  |
|  |  |  | 18568-18580 | (T)13 |  |  | * |  |  |  |  |  |  |  |  |  |  |  |  |  |  |  |  |  |  |  |
|  | Intron | *rpoC1* | 22993-23008 | (A)12,13,14,15,16 | (A)15 | (A)16 |  | (A)16 | (A)14 | (A)13 | (A)13 | (A)16 | (A)13 | (A)13 |  |  |  |  |  |  | (A)13 | (A)13 | (A)13 |  |  |  |
|  |  |  | 23015-23028 | (T)12 |  | * |  | * |  | * |  |  |  |  | * |  |  |  |  |  |  |  |  |  |  |  |
|  | IGS | *rpoB-trnC-GCA* | 28048-28080 | (A)12 |  | * |  |  |  |  | * | * | * | * |  |  |  |  |  |  |  |  |  |  |  |  |
|  | IGS | *petN-psbM* | 29550-29563 | (A)12,14 |  | (A)14 |  |  | (A)12 | (A)12 |  |  |  |  |  |  |  |  |  |  |  |  |  |  |  |  |
|  | IGS | *psbM-trnD-GUC* | 30072-30084 | (A)12 |  |  |  | * |  |  |  |  |  |  |  | * |  |  |  |  |  |  |  |  |  |  |
|  |  |  | 30127-30318 | (T)12 |  |  | * |  |  |  |  |  |  |  |  |  |  |  |  |  |  |  |  |  |  |  |
|  |  |  | 30358-30371 | (A)12 | * |  |  |  |  |  |  |  |  |  |  | * |  |  |  |  |  |  |  |  |  |  |
|  |  |  | 30742-30753 | (A)12 |  |  |  |  |  | * | * | * | * | * |  |  |  |  |  |  |  | * | * |  |  |  |
|  | IGS | *trnD-GUC-trnY-GUA* | 31288-31299 | (T)12 |  |  |  |  | * |  |  |  |  |  |  |  |  |  |  |  |  |  |  |  |  |  |
|  | IGS | *trnE-UUC-trnT-GGU* | 32019-32035 | (A)16 |  |  |  |  | * |  |  |  |  |  |  |  |  |  |  |  |  |  |  |  |  |  |
|  | IGS | *trnT-GGU-psbD* | 32638-32653 | (T)12,14 |  | (T)14 | (T)12 |  |  | (T)12 |  |  |  |  |  |  |  |  |  |  |  |  |  |  |  |  |
|  |  |  | 33128-33139 | (TCTT)3 | * | * | * | * | * | * | * | * | * | * |  |  |  |  |  |  |  |  |  |  |  |  |
|  | IGS | *trnG-GCC-trnfM-CAU* | 37604-37615 | (T)12 |  |  | * | * |  |  | * | * | * | * |  |  |  |  |  |  |  |  |  |  |  |  |
|  |  |  | 37622-37641 | (TCAAAA)3 |  |  |  |  |  |  |  |  |  |  | * |  | * | * | * | * |  |  |  |  |  |  |
|  |  |  | 37631-37646 | (AAAC)3 |  |  |  |  |  | * |  |  |  |  |  |  |  |  |  |  |  |  |  |  |  |  |
|  | IGS | *rps4-trnT-UGU* | 47256-47276 | (TA)6,7,9 |  | (TA)6 | (TA)6 | (TA)6 |  | (TA)6 | (TA)6 | (TA)6 | (TA)6 | (TA)6 | (TA)7 | (TA)7 | (TA)9 | (TA)9 | (TA)9 | (TA)9 | (TA)6 | (TA)6 | (TA)6 | (TA)6 | (TA)6 | (TA)6 |
|  |  |  | 47597-47608 | (A)12 |  |  |  |  |  |  |  |  |  |  | * | * |  |  |  |  |  |  |  |  |  |  |
|  |  |  | 47604-47619 | (AT)6,8 | (AT)6 |  |  | (AT)7 | (AT)6 | (AT)6 |  |  |  |  |  |  | (AT)8 | (AT)8 | (AT)8 | (AT)8 | (AT)6 |  |  |  |  |  |
|  |  |  | 47619-47632 | (TA)6,7 | (TA)6 | (TA)7 |  |  |  |  | (TA)7 | (TA)7 | (TA)7 | (TA)7 |  |  |  |  |  |  |  |  |  |  |  |  |
|  | IGS | *trnT-UGU-trnL-UAA* | 48127-48138 | (A)12 | * |  |  |  |  |  |  |  |  |  |  |  |  |  |  |  |  |  |  |  |  |  |
|  | Intron | *trnL-UAA* | 48605-48618 | (A)12,13,14 |  | (A)13 |  | (A)13 | (A)14 | (A)14 |  |  |  |  | (A)13 | (A)13 | (A)12 | (A)12 | (A)12 | (A)12 |  |  |  |  |  |  |
|  | IGS | *ndhK-ndhC* | 51542-51553 | (T)12 | * | * | * |  | * | * | * | * | * | * | * |  | * | * | * | * | * | * | * | * | * | * |
|  | IGS | *ndhC-trnV-UAC* | 52311-52322 | (TTTC)3 | * | * | * | * | * | * | * | * | * | * | * | * | * | * | * | * | * |  |  |  |  |  |
|  | IGS | *trnV-UAC-trnM-CAU* | 53534-53545 | (ATCT)3 | * | * | * |  | * | * | * | * | * | * | * | * | * | * | * | * | * | * | * | * | * | * |
|  | IGS | *trnM-CAU-atpE* | 53706-53721 | (T)14,15,16 | (T)15 | (T)15 | (T)15 | (T)14 | (T)15 | (T)15 | (T)15 | (T)15 | (T)15 | (T)15 | (T)15 | (T)16 | (T)15 | (T)15 | (T)15 | (T)15 | (T)15 | (T)15 | (T)15 | (T)16 | (T)15 | (T)15 |
|  | IGS | *atpB-rbcL* | 55745-55757 | (A)12,13 |  |  |  |  |  |  | (A)12 | (A)12 | (A)12 | (A)12 |  |  | (A)12 | (A)12 | (A)12 | (A)12 | (A)13 | (A)12 | (A)12 | (A)12 | (A)12 | (A)12 |
|  | IGS | *accD-psaI* | 60133-60157 | (T)12,15,16,17 |  |  |  |  |  |  |  |  |  |  |  |  | (T)12 |  |  |  | (T)15 | (T)15 | (T)15 | (T)16 | (T)17 | (T)16 |
|  |  |  | 60267-60281 | (A)15 | * |  |  |  |  |  |  |  |  |  |  |  |  |  |  |  |  |  |  |  |  |  |
|  | CDS | *psaI* | 60733-60744 | (TTTA)3 | * | * | * | * | * | * | * | * | * | * | * | * | * | * | * | * | * | * | * | * | * | * |
|  | IGS | *petL-petG* | 66751-66770 | (T)16,17,18,19,20 | (T)20 | (T)19 | (T)18 | (T)16 | (T)17 | (T)17 | (T)18 | (T)18 | (T)18 | (T)18 | (T)18 | (T)19 | (T)20 | (T)20 | (T)20 | (T)20 | (T)18 | (T)17 | (T)17 | (T)17 | (T)17 | (T)17 |
|  | IGS | *rpl33-rps18* | 68574-68586 | (T)13 |  |  |  |  |  |  | * | * | * | * |  |  |  |  |  |  |  |  |  |  |  |  |
|  |  |  | 68665-68679 | (TATTA)3 |  |  |  |  |  |  |  |  |  |  |  | * |  |  |  |  |  |  |  |  |  |  |
|  |  |  | 68678-68689 | (TA)6 |  |  |  |  |  |  | * | * | * | * |  |  |  |  |  |  |  |  |  |  |  |  |
|  | IGS | *rpl20-rps12* | 69940-69953 | (T)14 | * |  |  |  |  |  |  |  |  |  |  |  |  |  |  |  |  |  |  |  |  |  |
|  |  |  | 70437-70450 | (T)13 |  |  |  |  |  |  |  |  |  |  |  | * |  |  |  |  |  |  |  |  |  |  |
|  | Intron | *clpP Intron* | 71472-71483 | (AAAT)3 |  |  |  |  |  |  |  |  |  |  | * |  |  |  |  |  |  |  |  |  |  |  |
|  |  |  | 71537-71551 | (AAAAT)3 |  |  |  |  |  |  |  |  |  |  | * |  |  |  |  |  |  |  |  |  |  |  |
|  |  |  | 72068-72079 | (T)12 |  | * |  |  |  |  |  |  |  |  |  |  |  |  |  |  |  |  |  | * | * | * |
|  |  |  | 72227-72244 | (A)12,14,15,16 | (A)15 | (A)16 | (A)16 | (A)12 | (A)16 | (A)15 | (A)14 | (A)14 | (A)14 | (A)14 |  |  | (A)12 | (A)12 | (A)12 | (A)12 | (A)12 |  |  |  |  |  |
|  | IGS | *psbH-petB* | 75854-75865 | (TTTC)3 | * | * | * | * | * | * | * | * | * | * | * | * | * | * | * | * | * | * | * | * | * | * |
|  | Intron | *petD* | 77815-77828 | (AT)6,7 |  |  |  |  |  |  |  |  |  |  |  |  |  |  |  |  |  | (AT)6 | (AT)6 | (AT)7 | (AT)7 | (AT)7 |
|  | CDS | *rpoA* | 78957-78969 | (T)13 | * | * | * | * | * | * | * | * | * | * | * | * | * | * | * | * | * | * | * | * | * | * |
|  | IGS | *rps11-rpl36* | 80525-80536 | (T)12 |  |  |  |  |  |  |  |  |  |  | * | * | * | * | * | * |  | * | * | * | * | * |
|  | Intron | *rpl16* | 82886-82910 | (T)12,13,14,15 |  |  | (T)15 |  |  | (T)12 |  |  |  |  |  |  |  |  |  |  | (T)14 | (T)14 | (T)14 | (T)13 | (T)13 | (T)13 |
|  |  |  | 82950-82971 | (A)12,13,14 |  |  | (A)13 | (A)13 | (A)14 |  |  |  |  |  | (A)12 |  |  |  |  |  |  |  |  |  |  |  |
|  | CDS | *rpl22* | 85134-85147 | (T)14 | * | * | * | * | * |  | * | * | * | * | * | * | * | * | * | * | * | * | * | * | * | * |
| IRa | IGS | *rps19-rpl2* | 85622-85633 | (T)12 |  |  | * |  |  |  |  |  |  |  |  |  |  |  |  |  |  |  |  |  |  |  |
|  | CDS | *ycf2* | 90647-90658 | (CTT)4 | * | * | * | * | * | * | * | * | * | * | * | * | * | * | * | * | * | * | * | * | * | * |
|  | rRNA | *rrn23* | 105401-105412 | (AGGT)3 | * | * | * | * | * | * | * | * | * | * | * | * | * | * | * | * | * | * | * | * | * | * |
|  | IGS | *rrn4.5-rrn5* | 106884-106895 | (CCCT)3 | * | * | * | * | * | * | * | * | * | * | * | * | * | * | * | * | * | * | * | * | * | * |
| SSC | IGS | *ndhF-rpl32* | 112060-112074 | (T)13 |  |  | * |  |  |  |  |  |  |  |  |  |  |  |  |  |  |  |  |  |  |  |
|  |  |  | 112936-112948 | (AAT)4 |  |  |  |  |  | * |  |  |  |  |  |  |  |  |  |  |  |  |  |  |  |  |
|  | IGS | *rpl32-trnL-UAG* | 113619-113632 | (TA)6,7 |  |  |  |  |  |  | (TA)6 | (TA)7 | (TA)6 | (TA)6 |  |  |  |  |  |  | (TA)6 |  |  |  |  |  |
|  |  |  | 113867-113882 | (T)16 |  |  |  |  |  |  |  |  |  |  |  |  | * | * | * | * |  |  |  |  |  |  |
|  |  |  | 114113-114125 | (T)13 |  |  |  |  |  |  |  |  |  |  |  |  |  |  |  |  |  | * | * | * | * | * |
|  |  |  | 114129-114150 | (A)13,14,15 |  |  |  |  |  | (A)13 |  |  |  |  | (A)14 | (A)14 | (A)13 | (A)13 | (A)13 | (A)13 | (A)13 | (A)15 | (A)15 | (A)14 | (A)14 | (A)14 |
|  | IGS | *trnL-UAG-ccsA* | 114635-114650 | (T)12,13,14,15,16 |  |  |  |  |  |  |  |  |  |  | (T)12 | (T)13 | (T)15 | (T)14 | (T)14 | (T)14 | (T)14 | (T)16 | (T)16 | (T)16 | (T)16 | (T)16 |
|  | CDS | *ndhD* | 116051-116062 | (AATA)3 | * | * | * | * | * | * | * | * | * | * | * | * | * | * | * | * | * | * | * | * | * | * |
|  | IGS | *psaC-ndhE* | 117765-117782 | (TCTAGT)3 | * | * | * | * | * | * | * | * | * | * | * | * | * | * | * | * | * | * | * | * | * | * |
|  | CDS | *ndhE* | 118073-118095 | (TATT)3 |  | * | * |  |  |  |  |  |  |  | * | * | * | * | * | * | * | * | * | * | * | * |
|  | Intron | *ndhA* | 121028-121039 | (T)12 |  |  |  | * |  |  |  |  |  |  |  |  |  |  |  |  |  |  |  |  |  |  |
|  | IGS | *rps15-ycf1* | 127822-127833 | (TCTT)3 |  |  |  |  | * |  |  |  |  |  |  |  |  |  |  |  |  |  |  |  |  |  |
| IRb | IGS | *rrn4.5-rrn5* | 131038-131049 | (AGGG)3 | * | * | * | * | * | * | * | * | * | * | * | * | * | * | * | * | * | * | * | * | * | * |
|  | rRNA | *rrn23* | 132517-132528 | (CTAC)3 | * | * | * | * | * | * | * | * | * | * | * | * | * | * | * | * | * | * | * | * | * | * |
|  | CDS | *ycf2* | 147273-147284 | (AAG)4 | * | * | * | * | * | * | * | * | * | * | * | * | * | * | * | * | * | * | * | * | * | * |
|  | IGS | *rpl2-rps19* | 152298-152309 | (A)12 |  |  | * |  |  |  |  |  |  |  |  |  |  |  |  |  |  |  |  |  |  |  |

Notes: Except for “blo”, which stands for *A. blitoides*, and “blu”, which stands for *A. blitum*, the other species are represented by the first three letters of their species names. “*” indicates that SSR markers at this locus are the same in different species, and blank indicates that SSR markers at this locus are not present in this species.

**Table S3** The hotspot regions obtained based on the exhaustive method.

| **No.** | **Region** | **Gene/IGS** | **Length (bp)** | **SNP** | **Variant Frequency** |
| --- | --- | --- | --- | --- | --- |
| 1 | LSC | *trnK_rps16* | 1984 | 20 | 1.01% |
| 2 | LSC | *psbK_I+psbI+psbI_trnS+trnS+trnS_G* | 1788 | 14 | 0.78% |
| 3 | LSC | *atpA_F+atpF* | 1632 | 18 | 1.10% |
| 4 | LSC | *rpoC2* | 852 | 10 | 1.17% |
| 5 | LSC | *rpoB_trnC-GCA* | 1204 | 13 | 1.08% |
| 6 | LSC | *trnE-UUC_trnT-GGU+trnT+trnT_psbD* | 1040 | 11 | 1.06% |
| 7 | LSC | *psaA_ycf3+ycf3* | 1183 | 10 | 0.85% |
| 8 | LSC | *ycf3+ycf3_trnS* | 955 | 10 | 1.05% |
| 9 | LSC | *rps4_trnT-UGU+trnT+trnT-UGU_trnL-UAA* | 1677 | 25 | 1.49% |
| 10 | LSC | *trnF-GAA_ndhJ+ndhJ* | 899 | 10 | 1.11% |
| 11 | LSC | *psbE_petL+petL+petL_G* | 737 | 10 | 1.36% |
| 12 | LSC | *rpl16+rpl16_rps3* | 2617 | 26 | 0.99% |
| 13 | SSC | *ycf1+ndhF* | 2044 | 21 | 1.03% |
| 14 | SSC | *ndhF+ndhF_rpl32* | 2818 | 35 | 1.24% |
| 15 | SSC | *ndhD+ndhD_psaC+psaC+psaC_ndhE* | 1573 | 15 | 0.95% |
| 16 | SSC | *ycf1* | 2473 | 27 | 1.09% |

**Table S4** Materials information of *Amaranthus*.

| **Species** | **Provinces** | **collection numbers** |
| --- | --- | --- |
| *Amaranthus albus* | Xinjiang Urumchi | 2372 |
| *Amaranthus arenicola* | Jiangsu Taizhou | JSTZ |
| *Amaranthus arenicola* | Hebei Tangshan | HBTS |
| *Amaranthus blitoides* | Inner Mongolia Chifeng | 14 |
| *Amaranthus blitum* | Beijing | 2 |
| *Amaranthus capensis* | Jiangsu Taizhou | 12182 |
| *Amaranthus crispus* | Hebei Tangshan | 12128 |
| *Amaranthus deflexus* | Spain | 2011 |
| *Amaranthus dubius* | Shandong Qingdao | 12274 |
| *Amaranthus hybridus* | Zhejiang Hangzhou | 7 |
| *Amaranthus palmeri* | Beijing | 1 |
| *Amaranthus polygonoides* | Beijing | 90006 |
| *Amaranthus retroflexus* | Neimenggu Chifeng | 41 |
| *Amaranthus spinosus* | Beijing | 11902 |
| *Amaranthus spinosus* | Beijing | 113 |
| *Amaranthus standleyansus* | Zhejing Zhoushan | 7433 |
| *Amaranthus standleyansus* | Beijing | 11960 |
| *Amaranthus tunetanus* | Jiangsu Taizhou | 12127 |
| *Amaranthus tuberculatus var. rudis* | Jiangsu Taizhou | 12194 |
| *Amaranthus tuberculatus var. rudis* | Guangdong Xinsha | GZW |
| *Amaranthus tuberculatus var. tuberculatus* | Fujian Fuzhou | 11994 |

**Figure S1** The main insertion and deletion regions on SSC of *Amaranthus* were *ndhE-G* (77 bp) and *ndhG-I* (180 bp).

**
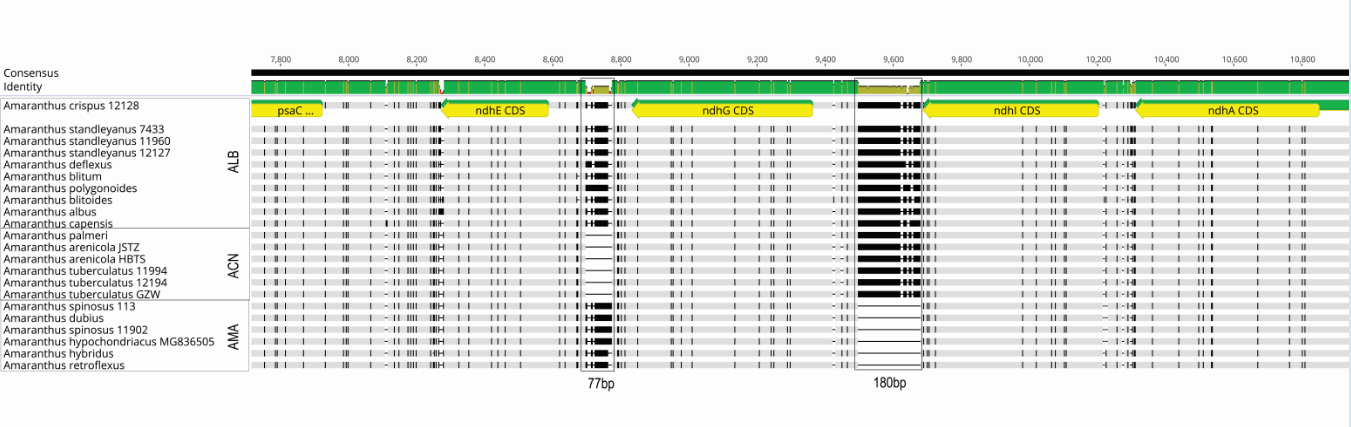
**Note: “AMA” represented the subgen. *Amaranthus*, “ACD” represented the subgen. *Acnida*, “ALB” represented the subgen. *Albersia*.
